# Temporal Variation in Population Size of European Bird Species: Effects of Latitude and Marginality of Distribution 

**DOI:** 10.1371/journal.pone.0077654

**Published:** 2013-10-17

**Authors:** José J. Cuervo, Anders P. Møller

**Affiliations:** 1 Department of Evolutionary Ecology, Museo Nacional de Ciencias Naturales, CSIC, Madrid, Spain; 2 Laboratoire d'Ecologie, Systématique et Evolution, CNRS UMR 8079, Université Paris-Sud, Orsay, France; University of Lausanne, Switzerland

## Abstract

In the Northern Hemisphere, global warming has been shown to affect animal populations in different ways, with southern populations in general suffering more from increased temperatures than northern populations of the same species. However, southern populations are also often marginal populations relative to the entire breeding range, and marginality may also have negative effects on populations. To disentangle the effects of latitude (possibly due to global warming) and marginality on temporal variation in population size, we investigated European breeding bird species across a latitudinal gradient. Population size estimates were regressed on years, and from these regressions we obtained the slope (a proxy for population trend) and the standard error of the estimate (SEE) (a proxy for population fluctuations). The possible relationships between marginality or latitude on one hand and slopes or SEE on the other were tested among populations within species. Potentially confounding factors such as census method, sampling effort, density-dependence, habitat fragmentation and number of sampling years were controlled statistically. Population latitude was positively related to regression slopes independent of marginality, with more positive slopes (i.e., trends) in northern than in southern populations. The degree of marginality was positively related to SEE independent of latitude, with marginal populations showing larger SEE (i.e., fluctuations) than central ones. Regression slopes were also significantly related to our estimate of density-dependence and SEE was significantly affected by the census method. These results are consistent with a scenario in which southern and northern populations of European bird species are negatively affected by marginality, with southern populations benefitting less from global warming than northern populations, thus potentially making southern populations more vulnerable to extinction.

## Introduction

There is currently general agreement that global warming is affecting animal and plant populations in multiple ways [1-4]. In temperate regions of the Northern Hemisphere, a northward shift in species distribution range has been detected [5-8], and this shift might be due not only to positive effects of increased temperatures on northern populations of a given species, but also to negative effects on southern populations. If that was the case, the latitude of a population would strongly determine the possible effects of increased temperatures on that population. We would expect a positive relationship between population trend and latitude, with positive trends at high latitudes and negative (or less positive) trends at low latitudes. In other words, populations close to the southern limit of the distribution of a species would be suffering to a greater extend (or benefiting less) from global warming than northern populations of the same species. Moreover, both southernmost and northernmost populations are also often marginal populations, and marginality (versus centrality) of a population across the distribution range may have a strong effect on fitness-related traits (e.g. developmental stability [9], predation rate [10], and reproductive success [11,12]) and thus potentially on population size variation. It is believed that marginal populations generally face worse environmental (biotic and/or abiotic) conditions than central populations [13,14]. In addition, marginal populations often have lower genetic variation than central populations [15-17], preventing or delaying marginal populations from adapting to extreme conditions. Because southernmost populations in temperate regions of the Northern Hemisphere would be particularly sensitive to both global warming and marginality, assessment of effects of global warming on these populations would require teasing apart the independent effect of the two phenomena. In general, marginal populations suffer greater fluctuations in abundance than central populations [18,19], and, therefore, a positive relationship between degree of fluctuation and marginality within species would be expected.

To disentangle the effects of latitude and marginality on temporal variation in population size we used European bird species as a case study. During recent years, the effects of climate change on bird species have been studied extensively, particularly in Europe. For example, we know that many bird species have experienced phenological changes paralleling the increase in temperature [20]. Such climate-driven changes may be due to phenotypic plasticity [21] or micro-evolutionary adaptations [22]. Importantly, the inability to provide appropriate phenological responses to climate changes has led to population declines in some species [23,24]. Distribution ranges have also changed in a number of bird species [25,26], with a northward move in agreement with predictions of habitat suitability changes due to global warming [27]. In the present study we investigated long-term trends in avian breeding populations in several European countries covering a wide latitudinal range. Taking into account variation in population size in different countries for every species, we were able to detect latitude-related differences in population changes. This approach has not been used so far, and it allowed us to make statistically powerful comparisons among populations (i.e., among countries) within species. As temperature varies with latitude, effects of global warming are predicted to differ among latitudes, and we were able to test whether latitudinal differences in population size variation were as predicted while simultaneously taking into account the effects of marginality.

Temporal variation in population size, and the causes and consequences of such variation, is a central topic in population biology (e.g. [28-32]), with clear implications for conservation biology [33,34]. Variation in any parameter is often measured as the coefficient of variation (CV), and this is also the case when estimating variation in population size. For example, we can estimate CV of population size among years. However, the CV of population size among years can be split into two different factors, in the same way as CV in the relative size of morphological traits [35,36]. If estimates of population size are regressed on years, high levels of among-year variation could arise either from large (positive or negative) slopes, from large dispersion of points around the regression line, i.e., a large standard error of the estimate (SEE), or both. These two components of variation provide qualitatively different information, and their independent effects should both be quantified. Slopes provide an estimate of population trends, with positive slopes for populations that are increasing in size, negative slopes for populations decreasing in size, and slopes close to zero when population size does not change over time. In contrast, SEE provides an estimate of population fluctuations, a factor that strongly affects the extinction risk of a population because larger fluctuations increase the probability that one of these reaches zero, i.e., extinction [37-39]. In addition, greater population fluctuations should also reduce genetic variation and hence the possibility for different species to adapt to changing environmental conditions (e. g. [32,40]). 

The aim of this study was to test the hypothesis that both latitude and marginality of the breeding distribution are independently related to temporal variation in population size. In within-species comparisons, we predicted (i) more positive population trends in northern than in southern populations, i.e., a positive relationship between slope and mean population latitude; and (ii) greater population fluctuations in marginal than in central populations, i.e., a positive relationship between SEE and population marginality.

## Methods

### Estimates of Population Size

Population size estimates of European bird species were obtained from websites and persons responsible for the Pan-European Common Bird Monitoring Scheme in every European country. We obtained data for only 12 countries, either because in some countries this scheme has started very recently or has not started yet, or because information was unavailable upon request. Population indices from some countries or regions could not be used in this study owing to incomplete information (e.g. Wallonia), or because bird censuses were done only in one type of habitat (e.g. Latvia). Countries with available and suitable information on avian population size, the source of this information, number of bird species per country, mean number of years with data in every country (not all species were always surveyed the same number of years in a country), and last year with information on population size are shown in Table 1. Canada goose *Branta canadensis* and common pheasant *Phasianus colchicus* were excluded because they are introduced in Europe [41,42]. In Sweden, information on the willow warbler only includes the subspecies *Phylloscopus trochilus acredula* and information on the chiffchaff only includes the subspecies *Phylloscopus collybita abietinus*. The search for information was finished by 8 June 2009. 

**Table 1:** Information used in this study concerning the 12 European countries with available estimates of population size (population indices) for avian species.

Country	Number of bird species	Mean number of years	Last year with population indices	Number of fieldworkers	Country area (km^2^)	Latitude (degrees)	Fragmentation index	Census method	Source of population indices
Austria	69	9	2006	145	83871	47.6965	2.295	Point-counts	Data provided by Norbert Teufelbauer
Czech Republic	103	25	2006	80	78866	49.8036	3.439	Point-counts	www.cso.cz/wpimages/other/TrendyJPSP82-06.pdf
Denmark	93	26.9	2004	300	43094	56.1557	4.497	Point-counts	Data provided by Dansk Ornithologisk Forening
Finland	84	23	2005	100	337030	64.9494	1.324	Point-counts and line transects	www.fmnh.helsinki.fi/seurannat/linjalaskenta/artikkelit/maalinnut/index.htm
France	170	14.6	2008	1000	535243	46.7111	3.560	Point-counts	www2.mnhn.fr/vigie-nature/ spip.php?page=stoc_web
Germany	100	15	2005	500	357104	51.0908	3.503	Point-counts, line transects and territory mapping	Data provided by Martin Flade
Hungary	91	9	2007	1000	93030	47.1613	4.044	Point-counts	Data provided by Tibor Szép
Netherlands	103	18	2007	500	41526	52.1078	3.636	Territory mapping	www.sovon.nl/xls/broedvogeltrends.xls
Norway	58	13	2007	120	324250	64.5560	1.425	Point-counts	Data provided by Magne Husby
Poland	102	7	2006	218	312679	51.9189	3.490	Line transects	www.otop.org.pl/upload/30/00/00/04/96/raport_mppl_2005-2006.zip
Spain	102	10.8	2008	890	496583	39.8953	2.920	Point-counts	www.seo.org/media/docs/ Graf_sp_98_08.pdf
Sweden	114	33.3	2008	175	449964	62.1983	1.527	Point-counts	www.zoo.ekol.lu.se/birdmonitoring/PDF-files/SomPKTforhomepage.xls

Country area is the area of the entire country, but in the case of France, Norway and Spain it does not include Corsica, Svalbard and Canary Islands, respectively. Fragmentation index for Norway is the mean of the Finnish and the Swedish values (see text for justification).

The estimates of population size that we obtained were always standardized to a value of one in a particular year and the rest of the years indicated a value relative to the reference year. For example, if the population size estimate in a year was two for a particular species, it meant that the population size of that species was twice the value in the reference year. Therefore, these population size estimates are always relative values and are usually called population indices (for an example of the use of population indices, see [43]). These indices were calculated in the same way in all European countries following recommendations made by the European Bird Census Council (EBCC; see http://www.ebcc.info). Specifically, population indices were calculated using the software TRIM (Trends and Indices for Monitoring Data). More information about the programme TRIM can be found at the website http://www.cbs.nl/en-GB/menu/themas/natuur-milieu/methoden/trim/default.htm. For most countries, the reference year was the first year of census, although that was not the case for Sweden, Germany and France, where the reference year was 1998, 1999 and 2001, respectively. In the case of Spain, the reference value was 0 instead of 1, and then population indices were transformed ((X+100)/100, where X was the population index), to make values comparable with those from other countries. For some countries (Czech Republic, Finland, France, and Poland) only graphs showing population indices, but not population indices themselves were available, and in these cases population indices were estimated from graphs using the program ImageJ (http://rsbweb.nih.gov/ij/). In order to assess the accuracy of population indices calculated from the graphs, we estimated population indices from graphs in two countries, Spain and Sweden, for which both population indices and graphs were available. Twenty five species were randomly chosen per country, but only considering species with data for the maximum number of years available for the country, i.e., 11 years in Spain and 34 years in Sweden. Population indices estimated from the graphs or directly shown at the websites were highly repeatable ([44]; Spain: *r* ≥ 0.998, *F*_10,11_ ≥ 842.00, *P* < 0.00001 for 25 species; Sweden: *r* ≥ 0.994, *F*_33,34_ ≥ 326.32, *P* < 0.00001 for 25 species).

The present study considered populations at the country level, a geographical scale considerably larger than the traditional ecological concept of a population, and thus we used countries as sample units. This should be appropriate for a continental-scale study, as was the case here, if two requirements are fulfilled. First, countries should be relatively small in relation to the breeding range of the birds, as happened to be the case here (mean (SE) country area = 0.263 x 10^6^ (0.053 x 10^6^) km^2^, *n* = 12 countries, Table 1; mean (SE) Western Palearctic breeding distribution area = 18.121 x 10^6^ (0.331 x 10^6^) km^2^, *n* = 73 bird species [32], and unpublished data). Second, censuses must be representative of the whole country, covering different regions and habitats within the country in a balanced way. This is the procedure recommended by the EBCC and followed by most countries. Other studies investigating gradients at a continental scale and population indices also used countries and/or very large regions as sample units (e. g. [45]).

Information on population indices is freely available in different websites (see Table 1) or can be provided upon request. 

### Population Latitude and Marginality

In order to estimate the marginality of bird populations, i.e., whether populations were central or marginal within the breeding distribution range, we first calculated a latitude index for each of the 12 European countries included in the study (Table 1). This latitude index was estimated as the latitude of the mid-point between the northernmost and the southernmost mainland points of every country. This information was obtained using maps in atlases and online with the software Google Earth (http://earth.google.com). Islands were not considered because they generally add a lot of distance in terms of latitudinal degrees, but very small area in relation to the entire country. The exception was Denmark, because a large part of that country is made up by islands, and the southernmost point of the country was located at Falster. The latitude index for each country was considered the latitude for all bird populations in that particular country regardless of the actual distribution of every species within a country. Once the latitude of the bird populations had been estimated, we calculated the distance (in degrees) from the bird population latitude to the northernmost and southernmost limits of the breeding distribution range of the species. The smaller of these two distances (L) was considered the smallest distance to the distribution limits of the species. In the few cases in which the country latitude index was more southern than the southernmost limit of the species range (herring gull *Larus argentatus* in France), or more northern than the northernmost limit of the species range (reed warbler *Acrocephalus scirpaceus* and golden oriole *Oriolus oriolus* in Finland, yellow-legged gull *Larus michahellis*, great spotted cuckoo *Clamator glandarius*, cattle egret *Bubulcus ibis*, southern grey shrike *Lanius meridionalis*, black-eared wheatear *Oenanthe hispanica*, subalpine warbler *Sylvia cantillans*, and Sardinian warbler *Sylvia melanocephala* in France, and woodlark *Lullula arborea* and red kite *Milvus milvus* in Sweden), the distance between bird population latitude and southernmost or northernmost limits of the distribution range of the species was considered to be zero. Northernmost and southernmost limits of the breeding distribution range of every species were obtained from published maps [46]. We also calculated the latitude of the mid-point between the northernmost and the southernmost limits of the distribution range of the species, and the distance (C) in degrees between this latitude and the bird population latitude. Marginality of a population was estimated as log_10_(C+1) - log_10_(L+1), with positive values representing marginal populations and negative values central populations. Finally, these values were transformed to ensure that marginality estimates ranged from 0 (central population) to 1 (marginal population). The transformation consisted of adding the absolute value of the most negative number and dividing by the largest value resulting from the previous addition. 

### Confounding Factors

One population parameter that might affect among-year variability in population size and, as a consequence, the results of our study is population density. One example of such an effect would be if populations at high density are close to carrying capacity. If that is the case, population size at high density is unlikely to vary much, and temporal variation in population size should be smaller than at low population density. However, populations at high density might vary to the same extent as populations at low density, the former mostly decreasing and the latter mostly increasing in size. In that case we would not expect a difference in temporal variation between populations with high and low density. Furthermore, in some populations with occasional or regular demographic explosions (e.g. snowy owl *Nyctea scandiaca*; [47]), variability in population size may be larger at high than at low density. Whatever happens in particular cases, density-dependence of population size variation is a general rule in a wide range of animal taxa [48-50]. Population indices do not provide information on absolute density (number of individuals per unit area), because they are relative values in relation to population size in a particular year. However, they give information about relative density within a population, because large population indices correspond to high density and small population indices to low density in that particular population. Therefore, within-population comparisons among years with (relatively) high and low density are possible. We estimated effects of density-dependence on among-year variability of population indices by comparing CV between years with high and low population indices (within populations). Specifically, for every bird population (i.e., every species in every country) we ranked population indices decreasingly and then divided the data in two halves, one with the largest population indices and the other with the smallest population indices. When number of study years was an odd number, the mid-point population index was included in both halves. In this way, the two subsets for a given population always included the same number of population indices. Then, CV of population indices was estimated for each of the two subsets, CV_high_ for large population indices and CV_low_ for small population indices. Since number of study years (n) used to calculate CV varied among countries and sometimes also among species within a country, CV values were transformed to obtain CV corrected for sample size (CV*) with the following transformation: CV* = CV (1 + 1/4n) [51]. Reduced Major Axis (RMA) regressions of CV*_high_ on CV*_low_ had in general slopes not significantly different from one after sequential Bonferroni correction ([52]; 12 tests; mean (SE) slope = 1.02 (0.05), *n* = 11, range from 0.79 to 1.25; -2.83 ≤ *t* ≤ 2.71, 56 ≤ df ≤ 168, and *P* ≥ 0.0078 for 11 countries, the exception being Poland: slope = 0.76, *t*_100_ = -3.65, and *P* < 0.001) and intercepts not significantly different from zero after sequential Bonferroni correction (mean (SE) intercept = -0.37 (0.59), *n* = 12, range from -4.47 to 2.39; -2.08 ≤ *t* ≤ 1.68, 56 ≤ df ≤ 168, and *P* ≥ 0.044 for the 12 countries). CV*_high_ and CV*_low_ did not differ significantly from each other after sequential Bonferroni correction when they were compared within populations (paired *t*-test; -2.60 ≤ *t* ≤ 1.72, 57 ≤ df ≤ 169, and *P* ≥ 0.012 for the 12 tests). As density-dependence should be controlled statistically, we included in our analyses a variable that reflected density-dependence in every population calculated as log_10_CV*_high_ - log_10_CV*_low_. Very large positive values would imply strong density-dependence with larger variation at high than at low densities. In contrast, very large negative values would indicate strong density-dependent effects with larger variation at low than at high densities. Values around zero would imply weak or no density-dependence.

Another parameter that might have an effect on population size is habitat fragmentation [53-55]. Fragmentation of land due to urbanisation, transport infrastructure and agriculture has been estimated for most European Union countries by the European Environment Agency (EEA). Methods used by EEA to calculate fragmentation indices and a map of Europe at a 10x10 km grid resolution showing levels of fragmentation can be found at the website http://www.eea.europa.eu/data-and-maps/figures/fragmentation-by-urbanisation-infrastructure-and-agriculture. In this map, the degree of fragmentation is shown by different colours representing six categories of fragmentation from minimal to extreme. We first calculated the percentage of the area of each country for every category of fragmentation by using the software Adobe Photoshop CS4 Extended, v. 11.0.2 (Adobe Systems Inc., San Jose, CA). We then assigned a value of fragmentation from one to six to the different degrees of fragmentation and calculated the mean fragmentation index weighted by area for every country (Table 1). Information on fragmentation was unavailable for Norway, but we assumed that it was very similar to the fragmentation index of the other countries of the Scandinavian Peninsula, i.e., Finland and Sweden. We performed all the statistical analyses three times using as fragmentation index for Norway the Finnish value, the Swedish value, or the mean of the Finnish and Swedish values. As results were qualitatively identical in the three cases, for brevity we only present results obtained using the mean of the Finnish and Swedish values. 

 At least two methodological factors might affect the estimation of population indices. First, we would expect that sampling effort affected population size estimates, with possible implications for among-year variability of estimates. Sampling effort was calculated as the number of fieldworkers in a country (this information can be found at the EBCC website, see Table 1) divided by area of the country (Table 1), i.e., fieldworkers per square kilometre. Second, census method might influence estimates of population fluctuations if some methods are more prone to errors than others. Actually, this seems to be the case, especially because point-counts show particularly large errors (B.-E. Sæther, personal communication). Observation errors have been suggested to significantly affect not only estimates of variation in population size [50], but also the apparent influence of density-dependence on this variation [56]. According to the EBCC website, most countries only used point-counts in their censuses, but methodology differed in four countries (Table 1). To control for different methodologies in different countries, we included in the analyses the variable “census method” with two categories, countries exclusively using point-counts (eight countries) and countries using other methods (four countries).

 The present study implicitly assumes that global warming is both geographically and temporally uniform, allowing comparisons between time series of 34 years (Sweden) and only seven years (Poland), or between southern and northern European populations. Although these assumptions might not generally hold, we consider them to be acceptable in our case for the following reasons. Regarding geographic variation, almost all 12 countries included in this study have experienced a temperature increase during the twentieth century (range from -0.05°C to 1.27°C, mean (SE) = 0.67 (0.12) °C; temperature data obtained from [57]), while the relationship between temperature change and latitude for the 12 countries is far from significant (Pearson correlation, *r* = -0.250, *n* = 12, *P* = 0.43). In relation to temporal variation, and using data from the EEA (http://www.eea.europa.eu), the relationship between annual temperature change in Europe during 1975-2008 and year is also not statistically significant (Pearson correlation, *r* = 0.074, *n* = 34, *P* = 0.68). These results suggest that warming has been similar in southern and northern Europe and constant over the 34 years considered in the present study. We here use temperature variation as a proxy for climate variation that is necessarily more complex than just differences in temperature.

### Statistical Analyses

The hypothetical relationships between marginality or latitude and population trends or fluctuations were tested among populations of every species. We obtained information on population indices and marginality for a total of 231 bird species, although the number of countries with information for every species differed greatly. For 55 species we could only find information in one country, for 45 species in two countries, 16 species in three countries, 14 species in four countries, five species in five countries, six species in six countries, 16 species in seven countries, ten species in eight countries, 12 species in nine countries, 15 species in ten countries, 15 species in 11 countries, and 22 species in all 12 countries. Since we were interested in testing for relationships among populations within species (i.e., among countries within species), it would make no sense to include in the analyses species with information for only one or two countries. On the other hand, if only species with information for all 12 countries were included, sample size (number of species) would be dramatically reduced, consequently decreasing statistical power. Therefore, we attempted to find a compromise between these extremes, including as many species as possible but only species with information for a reasonable number of countries. We chose species with data for at least eight countries (74 species) because this allows sufficient variation among populations of the same species, while simultaneously maintaining a relatively large sample size. However, we repeated the analyses including species with data for at least seven countries (90 species) and including only species with data for at least nine countries (64 species). In general, qualitatively similar results were obtained with the three data sets, with virtually no difference in the effect of our variables of interest, i.e., population marginality and latitude (see Appendix S1 in Supporting Information). The only qualitative difference referred to the relationship between SEE and habitat fragmentation (see Appendix S1).

**Figure 1:**
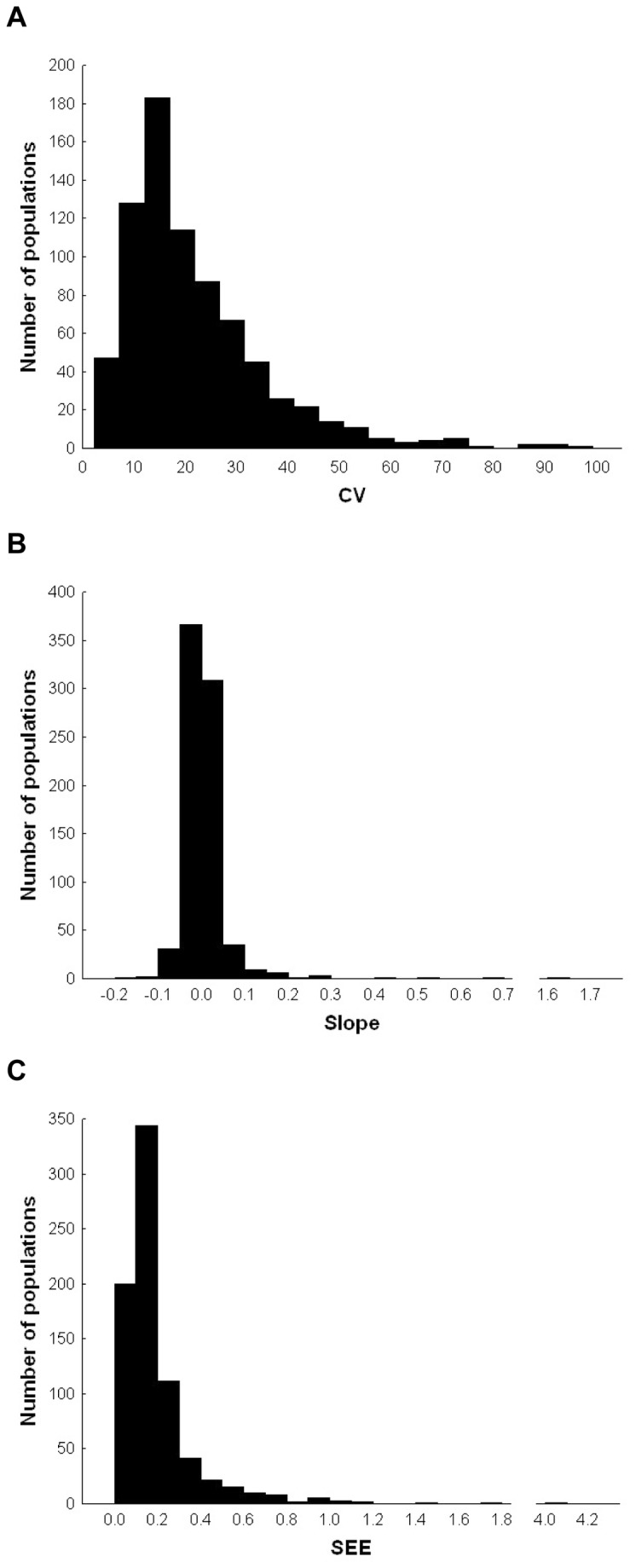
Frequency distribution of population parameters in 767 populations of 74 European breeding bird species. (a) Coefficient of variation (CV) of population size estimates (population indices) corrected for sample size (see text); (b) slope and (c) standard error of the estimate (SEE) after regressing population indices on year. Outliers are included (see text). Mean (SE) for the three parameters is CV: 21.95 (0.51); slope: 0.008 (0.003); SEE: 0.202 (0.008).

 To test for the hypothetical effect of marginality or latitude of a population on among-year variability in population size of European birds we performed a General Linear Mixed Model (GLMM), with CV of population indices among years (corrected for sample size, see above) as the dependent variable and marginality (as defined above) and latitude of the population (in degrees) as predictor variables. Species was included as a random factor and, as a result, all putative relationships between dependent variables (e.g. CV) and predictors (e.g. latitude) were tested within species, i.e., among populations of the same species. We also included in the model the density-dependence estimate, sampling effort, census method, habitat fragmentation (see estimate or definition of these four variables above), and number of years with population indices. As explained in the Introduction, among-year variability in population indices can be split into two components, namely the slope of the regression of population indices on years, and the dispersion of points around the slope, i.e., SEE of the regression. Therefore, we regressed population indices on years for every population, and repeated the same GLMM analysis described above, but with slope or SEE of these regressions as dependent variables. We noticed that two populations were clear outliers, namely the slope for stock pigeon *Columba oenas* in Hungary, which was 1.57 (all other slopes ranged from -0.18 to 0.67), and SEE for coot *Fulica atra* in Hungary, which was 4.01 (all other SEE ranged from 0.02 to 1.76) (Figure 1). Thus, we excluded these two populations from the analyses, implying exclusion of all coot data, because when excluding one population, the coot was no longer a species with data for eight countries. Analyses including the outliers in general yielded similar results, especially regarding population marginality and latitude (see Results). 

 In addition to testing for a possible relationship between latitude and population trend, we also estimated whether trends were positive or negative for southern and northern populations. First, all populations within each species were divided in two halves, the northernmost and southernmost populations, and the mean slope was calculated for each half. When the number of populations was an odd number, the most central population was excluded from the analyses. Second, slope means for northern and southern populations were averaged across species.

We acknowledge that slopes after regressing population indices on years are rough estimates of population trends (for more refined methods, see e.g. [58,59]). However, we are mainly interested in relative trends among populations (within species). As all slopes have been calculated in the same way, comparisons among them should be appropriate. Intrinsic variation associated with the estimates of annual population indices was not taken into account because it was unavailable for most countries. Nevertheless, as factors potentially affecting variation in these estimates (e.g., habitat fragmentation, sampling method or sampling effort) have been controlled in the analyses, we assume that the remaining variation is random (unbiased) and will add only noise to our analyses, making any significant relationship between variables conservative. 

 Statistical analyses in this study implicitly assume that population parameters in different countries are independent. However, this assumption might not be met if population parameters tend to be more closely related in neighbouring than in more distant countries. To address this issue, we checked whether CV, slope and SEE were more strongly correlated in contiguous than in non-contiguous countries. We considered contiguous countries to be those that shared a land border. In our case, there was a maximum of 13 pairs of contiguous countries (e.g., Germany-Austria, Germany-Poland, Spain-France, Norway-Finland, etc.). For each bird species, we correlated the three population parameters between all pairs of contiguous countries and also between the same number of pairs of non-contiguous countries. Pairs of non-contiguous countries were chosen randomly except for the fact that we only included countries already present in the pairs of contiguous countries for that species. Mean Pearson correlation coefficients across the 73 bird species were 0.0002 (CV), 0.083 (slope) and 0.012 (SEE) for contiguous countries and -0.079 (CV), -0.136 (slope) and -0.085 (SEE) for non-contiguous countries. In none of the three cases was the absolute value of the mean correlation coefficient larger in contiguous than in non-contiguous countries. This implies that population parameters were not more closely related in nearby than in more distant countries.

The number of years with population indices varied greatly among countries (Table 1), and we partially controlled for this variation by including the number of years as a covariate in the models. However, these analyses implicitly assume that population parameters are relatively constant through time. We checked this assumption in the four countries with data for more than 20 years (Czech Republic, Denmark, Finland and Sweden) by calculating CV, slope and SEE for every species in the last 10 years and in the previous 10 years, checking whether the two sets of data were related. In GLMM analyses including country as a random factor, we found positive and significant relationships between decades for the three population parameters (*F*_1,254_ ≥ 11.49, *P* < 0.001 in the three cases). This means that population changes for a given species were similar in different decades, at least in these four countries. In some countries (Austria, Hungary and Poland), the number of years with population indices was rather small (< 10; Table 1), making population parameters more prone to error and thus less reliable. However, these countries did not have a strong influence on the results, because exclusion of them from the analyses generally provided qualitatively similar results (Appendix S1). The most remarkable difference was that the relationship between slope and latitude was statistically significant only after removal of non-significant factors from the model (see Appendix S1).

All statistical analyses were two-tailed with a significance level of 0.05, and performed with Stastistica v. 9.0 (http://www.statsoft.com), except RMA regressions that were performed with RMA Software for Reduced Major Axis Regression v. 1.17 (http://www.bio.sdsu.edu/pub/andy/RMA.html).

An example of how population parameters were calculated in a common bird species (the great tit *Parus major*) can be found in Appendix S2 (Supporting Information).

## Results

Population latitude was significantly positively related to the slope of the relationship between population size and year, but not to CV or SEE (Table 2). The relationship between population latitude and slope indicates that population trends were more positive for northern than for southern populations. While northern populations generally increased in size over time (mean slope (SE) = 0.0079 (0.0032); one-sample *t*-test against zero; *t*_72_ = 2.48, *P* = 0.015), southern populations did not change significantly in size during the same period (mean slope (SE) = 0.0030 (0.0037); *t*_72_ = 0.83, *P* = 0.41). In contrast, marginality of populations, i.e., whether populations were close to the latitudinal limits of distribution of the species (marginal populations) or close to the centre of that distribution (central populations), was positively and significantly related to CV, and this association was due to the relationship with SEE, while it was not significantly related to the slope (Table 2). Specifically, marginal populations showed larger dispersion of observations around the regression line and hence larger among-year variability than central populations. However, the degree of marginality was not significantly related to the trend of the population (Table 2). 

**Table 2:** General Linear Mixed Models with among-year variability in population size (CV of population indices corrected for sample size, see text), and slope and standard error of the estimate (SEE) after regressing population indices on year as dependent variables.

Dependent variables	Independent variables	Sum of squares	df	*F*	*P*	Beta (SE)
CV	Species	32290.13	72	3.22	< 0.0001	
	Census method	3206.62	1	23.05	< 0.0001	
	Sampling effort	185.84	1	1.34	0.25	-0.045 (0.039)
	Density-dependence	710.94	1	5.11	0.024	0.074 (0.033)
	Number of years	2415.80	1	17.37	< 0.0001	0.172 (0.041)
	Habitat fragmentation	2080.18	1	14.95	0.00012	0.203 (0.052)
	Latitude	95.63	1	0.69	0.41	-0.057 (0.069)
	Marginality	3323.55	1	23.89	< 0.0001	0.395 (0.081)
	Error	94310.09	678			
Slope	Species	0.379	72	2.35	< 0.0001	
	Census method	0.003	1	1.48	0.22	
	Sampling effort	0.006	1	2.71	0.10	0.070 (0.043)
	Density-dependence	0.048	1	21.19	< 0.0001	-0.164 (0.036)
	Number of years	0.007	1	2.91	0.088	-0.077 (0.045)
	Habitat fragmentation	0.002	1	0.80	0.37	-0.051 (0.057)
	Latitude	0.013	1	6.00	0.015	0.185 (0.076)
	Marginality	0.007	1	3.09	0.079	-0.155 (0.088)
	Error	1.520	678			
SEE	Species	4.889	72	2.89	< 0.0001	
	Census method	0.684	1	29.09	< 0.0001	
	Sampling effort	0.041	1	1.74	0.19	-0.053 (0.040)
	Density-dependence	0.001	1	0.05	0.82	-0.008 (0.034)
	Number of years	0.088	1	3.73	0.054	0.082 (0.042)
	Habitat fragmentation	0.108	1	4.58	0.033	0.115 (0.054)
	Latitude	0.002	1	0.08	0.78	0.020 (0.071)
	Marginality	0.369	1	15.69	< 0.0001	0.329 (0.083)
	Error	15.953	678			

Species (random factor), census method (fixed factor), sampling effort, density-dependence, number of years surveyed, habitat fragmentation, population latitude and marginality were included in the model as independent variables. For definition and calculation of variables, see text. Full models had the statistics: CV, *F*_79,678_ = 4.66, *r* ^2^ = 0.352, *P* < 0.0001; slope, *F*_79,678_ = 2.50, *r* ^2^ = 0.226, *P* < 0.0001; SEE, *F*_79,678_ = 3.95, *r* ^2^ = 0.315, *P* < 0.0001.

 Regarding the other parameters that hypothetically might have an effect on CV, SEE or slope, most of them were significantly related to at least one dependent variable. Census method had a significant effect on CV and SEE (Table 2), with both variables showing larger values when exclusively point-counts had been used to estimate population size (CV: point-counts, least squares (LS) mean (SE) = 23.90 (0.57), *n* = 494, other methods, LS mean (SE) = 18.87 (0.81), *n* = 264; SEE: point-counts, LS mean (SE) = 0.224 (0.007), other methods, LS mean (SE) = 0.150 (0.010)). The effect of census method on the slope was not statistically significant (Table 2). Sampling effort was not significantly related to CV, slope or SEE (Table 2, but see results below when outliers were included in the analyses). Significant density-dependent effects were found for CV, mainly because of the negative relationship between density-dependence and slope (Table 2). Large positive slopes occurred when population indices varied more at low than at high densities, and large negative slopes when population indices varied more at high than at low densities. Fragmentation was significantly related to CV and SEE (Table 2), with highly fragmented habitats associated with high levels of among-year variation in population size. Finally, the number of years was significantly related to CV (Table 2), with populations surveyed for many years showing larger CV than populations surveyed for few years.

 The analyses performed after including outliers (see Figure 1 for visualization of outliers and Methods for further explanations) provided qualitatively identical results in relation to population latitude and marginality (see Appendix S1), although there were differences for other variables. First, inclusion of outliers yielded a significant relationship between slope and number of study years (beta (SE) = -0.102 (0.046), *F*_1,686_ = 4.93, *P* = 0.027). Second, the relationship between habitat fragmentation and SEE was no longer significant, a similar result to that obtained when including only species with data for at least seven or nine countries (see Appendix S1). Third, the slope was significantly related to sampling effort when outliers were included (beta (SE) = 0.105 (0.044), *F*_1,686_ = 5.78, *P* = 0.016).

 We considered that the relationship between two variables was robust only when all analysed subsets of data provided qualitatively similar results, i.e., when the relationship was statistically significant in all cases. With this criterion, we found that the slope (i.e., the population trend) was only significantly related to latitude and the index of density-dependence. In contrast, SEE (i.e., population fluctuations) was significantly related to population marginality and census method. Finally, CV of population indices was significantly related to population marginality, census method, density-dependence, habitat fragmentation, and number of years surveyed. In cases in which mixed results were obtained with different subsets of data, we considered that our study did not provide support for accepting or rejecting the null hypothesis.

## Discussion

The main result of this study was the significant relationship between latitude and population trend, once marginality and other confounding factors had been controlled statistically. Northern populations of European bird species showed more positive trends than southern populations, as expected if climate warming had an effect on population size. Our study is therefore consistent with the conclusion reached in previous studies using other approaches or investigating other taxa [3,4,45,60], namely that climate change is having a non-negligible effect on population trends, and that this effect is more beneficial for northern than for southern populations. We are aware that the relationship between latitude and population trend does not necessarily imply an effect of climate warming, because any other latitude-related factor might be responsible for such an association. However, the large amount of literature suggesting an effect of climate change on animal populations (see references above and in the Introduction), together with the fact that some factors potentially affecting population trend (e.g. density-dependence or habitat fragmentation) were already taken into account, make global warming a likely candidate to explain our result.

Until now, most studies investigating the effect of global warming on population trends have focused on changes in the range of the distribution, either latitudinal or altitudinal (e.g. [5,61]), and fewer studies have focused on changes in population size (e.g. [62,63]). The present study used a direct and statistically powerful approach focusing on latitudinal variation in population trends within species. This approach has not been used before, possibly because of difficulties in obtaining population size estimates for different populations of the same species. Inclusion in this study of population indices of European birds covering many species and populations was possible because (i) birds are one of the best studied classes of animals, particularly in Europe, with thousands of professional and non-professional birders surveying bird populations, (ii) international continent-wide programmes such as the Pan-European Common Bird Monitoring Scheme provide the opportunity to standardize methodologies and share resources, and (iii) the information obtained by this programme is often freely accessible. 

 Another important result of our study was the predicted positive relationship between population fluctuations and marginality, once the effects of latitude and other confounding factors had been controlled. Marginality was defined as the distance from the population latitude to the closest (northern or southern) limit of the breeding distribution range of the species relative to the distance to the centre of the breeding distribution range. Ecological conditions at the margin were most likely suboptimal for that particular species compared to conditions at the centre of the distribution range [13,64,65]. The bird species included in this study fluctuated more widely at the edges of their distribution range and, as explained in the Introduction, the larger the fluctuation the higher the risk of extinction of a particular population. Therefore, southern and northern populations of European bird species suffered greater fluctuations than central populations because of their marginality. 

According to our results, northern populations of European birds experienced an increase in size, while southern populations did not experience such an increase. In addition, both southern and northern populations suffered greater fluctuations than central populations. Therefore, southern populations might be particularly vulnerable to extinction: they fluctuate greatly because they are marginal populations, but do not benefit from global warming because they are living at low latitudes for the species. These two factors, population trend and fluctuations, could even interact, because a reduction in population size may cause a further increase in population fluctuations [66,67] and consequently also an increase in the risk of extinction. Moreover, if local extinctions mainly occur in southern populations, such populations will experience greater fragmentation that in turn may contribute to accelerate the extinction of the remaining southern populations [68,69].

Variations in population size may have significant genetic consequences. Nagylaky [70] and Wakeley [71] showed that the number of heterozygous loci under certain assumptions is a function of effective population size and mutation rate. Published estimates of effective population size only exist for a couple of the species included in our study. However, species that differ little in population fluctuations and continuously have large populations should be able to maintain greater levels of genetic variation as shown for the species included in the present study [32]. The positive relationship between genetic variation and population size implies that a long-term decline in population size will reduce the level of genetic variation that in turn will increase the risk of extinction [72].

 Both density-dependent effects and census methods were significantly related to temporal variation in population size. First, population trends were not only related to latitude but also to density-dependence. When populations fluctuated more markedly at low than at high densities, these high densities were probably close to carrying capacity. In these circumstances, bird populations showed more positive trends. This means that populations that increased in size might be close to carrying capacity at the end of the surveys. In contrast, the larger the population fluctuation at high compared to low densities, the further such high densities probably were from carrying capacity. These populations showed more negative trends, implying that they might have already been far from carrying capacity when surveys began, and decreased in size even more during the following years. It can be deduced from these results that populations either increasing or decreasing considerably in size were generally far from carrying capacity when surveys started. Moreover, as northern populations increased in size more than southern populations (see Results), northern populations should be closer to carrying capacity than southern populations at the end of the surveys. Second, population fluctuations were significantly related to census method, because our estimates of population fluctuations were larger in countries in which point-counts exclusively had been used to estimate population size. One possible explanation for this result is that point-count methods are more prone to observer errors than other census methods, thus giving rise to larger estimates of variation in population size. 

 Interestingly, temporal variation in population size always differed significantly among species (see Table 2 and Appendix S1). If populations of some species vary more markedly in size than others we can speculate that certain ecological, life-history or genetic characteristics of the species will be related to the degree of variation. Any predictions relating population fluctuations to ecology of different species are so far generally untested.

In conclusion, the results of this study are consistent with the hypothesis that climate change is having a substantial effect on population size of European bird species, and southern populations are suffering from large fluctuations because of marginal distribution but do not benefit from global warming. This has significant implications for conservation strategies, and southern populations of European bird species should be priority targets of conservation measures, especially species with local population differentiation hence representing unique biological diversity.

## Supporting Information

Appendix S1 Results when a different subset of bird species or countries was included in the analyses.
(DOC)

Appendix S2 An illustrative example of calculation of population parameters for a common bird: the great tit.
(DOC)
