# Supplementary material for: Temporal Variation in Population Size of European Bird Species: Effects of Latitude and Marginality of Distribution
Source: PLoS One. 2013 Oct 17;8(10):e77654. doi: 10.1371/journal.pone.0077654 (PMC3798344; doi:10.1371/journal.pone.0077654)
Supplement: Appendix S1 — Results when a different subset of bird species or countries was included in the analyses. (DOC) [file pone.0077654.s001.doc]

**Appendix S1.** Results when a different subset of bird species or countries was included in the analyses

When a different subset of species was included in the analyses (species with data for at least seven countries or species with data for at least nine countries), most results were qualitatively identical, particularly the relationships between population latitude or marginality and CV, SEE and slope (Table S1). This means that our results are robust and do not depend on the specific number of species included in the analyses. There was only one important difference. The relationship between habitat fragmentation and SEE was statistically significant when including species with data for at least eight countries, but was not significant when including only species with data for at least nine (*F*_1,615_ = 3.44, *P* = 0.064) or seven countries (*F*_1,780_ = 2.74, *P* = 0.098). However, the positive relationship between habitat fragmentation and CV remained significant in the three subsets of bird species (Table S1).

When countries with less than ten years of population indices (Austria, Hungary and Poland) were excluded from the analyses, most results for population latitude and marginality were qualitatively identical, although the relationship between slope and latitude was statistically significant only after backward stepwise removal of non-significant (*P* ≥ 0.10) factors from the model (Table S2). Marginality of population was retained in the reduced final model because we were interested in checking the possible relationship between slope and population latitude while simultaneously taking into account the effect of marginality. In any case, the relationship between slope and latitude was still statistically significant (*F*_1,494_ = 4.55, *P* = 0.033) if marginality of the population was removed from the model.

**Table S1.** General Linear Mixed Models including different subsets of species, with inter-year variability in population size (CV of population indices corrected for sample size), and slope and standard error of the estimate (SEE) after regressing population indices on year as dependent variables.

| Dependent variables | Independent variables | Including species with data for at least 7 countries | | | Including species with data for at least 9 countries | | | Including species with data for at least 8 countries but including the outliers | | |
| --- | --- | --- | --- | --- | --- | --- | --- | --- | --- | --- |
|  |  | df | *F* | *P* | df | *F* | *P* | df | *F* | *P* |
| CV | Species | 89 | 3.64 | < 0.0001 | 63 | 2.95 | < 0.0001 | 73 | 3.31 | < 0.0001 |
|  | Census method | 1 | 32.02 | < 0.0001 | 1 | 20.49 | < 0.0001 | 1 | 25.46 | < 0.0001 |
|  | Sampling effort | 1 | 0.50 | 0.48 | 1 | 0.62 | 0.43 | 1 | 0.72 | 0.40 |
|  | Density-dependence | 1 | 6.34 | 0.012 | 1 | 7.31 | 0.0071 | 1 | 4.50 | 0.034 |
|  | Number of years | 1 | 14.86 | 0.00013 | 1 | 15.39 | < 0.0001 | 1 | 15.55 | < 0.0001 |
|  | Habitat fragmentation | 1 | 10.53 | 0.0012 | 1 | 11.60 | 0.00070 | 1 | 14.50 | 0.00015 |
|  | Latitude | 1 | 0.06 | 0.80 | 1 | 1.04 | 0.31 | 1 | 0.54 | 0.46 |
|  | Marginality | 1 | 13.86 | 0.00021 | 1 | 24.65 | < 0.0001 | 1 | 24.07 | < 0.0001 |
|  | Error | 780 |  |  | 615 |  |  | 686 |  |  |
| Slope | Species | 89 | 1.94 | < 0.0001 | 63 | 2.38 | < 0.0001 | 73 | 1.71 | 0.00042 |
|  | Census method | 1 | 0.60 | 0.44 | 1 | 0.55 | 0.46 | 1 | 3.51 | 0.061 |
|  | Sampling effort | 1 | 1.23 | 0.27 | 1 | 1.78 | 0.18 | 1 | 5.78 | 0.016 |
|  | Density-dependence | 1 | 23.20 | < 0.0001 | 1 | 18.76 | < 0.0001 | 1 | 14.50 | 0.00015 |
|  | Number of years | 1 | 2.81 | 0.094 | 1 | 1.65 | 0.20 | 1 | 4.93 | 0.027 |
|  | Habitat fragmentation | 1 | 0.03 | 0.86 | 1 | 0.86 | 0.35 | 1 | 0.32 | 0.57 |
|  | Latitude | 1 | 4.49 | 0.034 | 1 | 4.32 | 0.038 | 1 | 3.94 | 0.048 |
|  | Marginality | 1 | 1.34 | 0.25 | 1 | 2.67 | 0.10 | 1 | 1.61 | 0.21 |
|  | Error | 780 |  |  | 615 |  |  | 686 |  |  |
| SEE | Species | 89 | 2.76 | < 0.0001 | 63 | 3.12 | < 0.0001 | 73 | 2.92 | < 0.0001 |
|  | Census method | 1 | 23.96 | < 0.0001 | 1 | 29.44 | < 0.0001 | 1 | 23.74 | < 0.0001 |
|  | Sampling effort | 1 | 2.10 | 0.15 | 1 | 0.68 | 0.41 | 1 | 0.03 | 0.87 |
|  | Density-dependence | 1 | 0.02 | 0.88 | 1 | 0.01 | 0.90 | 1 | 0.37 | 0.54 |
|  | Number of years | 1 | 2.83 | 0.093 | 1 | 3.15 | 0.077 | 1 | 0.33 | 0.56 |
|  | Habitat fragmentation | 1 | 2.74 | 0.098 | 1 | 3.44 | 0.064 | 1 | 3.01 | 0.083 |
|  | Latitude | 1 | 1.87 | 0.17 | 1 | 0.001 | 0.98 | 1 | 0.06 | 0.80 |
|  | Marginality | 1 | 4.15 | 0.042 | 1 | 16.08 | < 0.0001 | 1 | 16.27 | < 0.0001 |
|  | Error | 780 |  |  | 615 |  |  | 686 |  |  |

Species (random factor), census method (fixed factor), sampling effort, density-dependence, number of years surveyed, habitat fragmentation, population latitude and marginality were included in the models as independent variables. Every model included a different subset of bird populations. In all nine cases the full model was highly significant (*F* ≥ 1.94, 70,615 ≤ df ≤ 96,780, *r*^2^ ≥ 0.185, *P* < 0.0001). See Methods for further details.

**Table S2.** General Linear Mixed Models including a different subset of countries, with inter-year variability in population size (CV of population indices corrected for sample size), and slope and standard error of the estimate (SEE) after regressing population indices on year as dependent variables.

| Dependent variables | Independent variables | Excluding countries with data for less than 10 years | | | Excluding countries with data for less than 10 years and removing non-significant factors from the model (see the text for details) | | | | | |
| --- | --- | --- | --- | --- | --- | --- | --- | --- | --- | --- |
|  |  | df | *F* | *P* | df | *F* | *P* |  |  |  |
| CV | Species | 72 | 2.64 | < 0.0001 |  |  |  |  |  |  |
|  | Census method | 1 | 13.83 | 0.00022 |  |  |  |  |  |  |
|  | Sampling effort | 1 | 1.50 | 0.22 |  |  |  |  |  |  |
|  | Density-dependence | 1 | 3.32 | 0.069 |  |  |  |  |  |  |
|  | Number of years | 1 | 11.89 | 0.00061 |  |  |  |  |  |  |
|  | Habitat fragmentation | 1 | 5.90 | 0.016 |  |  |  |  |  |  |
|  | Latitude | 1 | 0.88 | 0.35 |  |  |  |  |  |  |
|  | Marginality | 1 | 15.97 | < 0.0001 |  |  |  |  |  |  |
|  | Error | 489 |  |  |  |  |  |  |  |  |
| Slope | Species | 72 | 2.20 | < 0.0001 | 72 | 2.20 | < 0.0001 |  |  |  |
|  | Census method | 1 | 1.12 | 0.29 | - | - | - |  |  |  |
|  | Sampling effort | 1 | 3.35 | 0.068 | - | - | - |  |  |  |
|  | Density-dependence | 1 | 31.63 | < 0.0001 | 1 | 32.30 | < 0.0001 |  |  |  |
|  | Number of years | 1 | 0.06 | 0.81 | - | - | - |  |  |  |
|  | Habitat fragmentation | 1 | 2.87 | 0.091 | - | - | - |  |  |  |
|  | Latitude | 1 | 2.37 | 0.12 | 1 | 4.40 | 0.036 |  |  |  |
|  | Marginality | 1 | 2.25 | 0.13 | 1 | 1.25 | 0.26 |  |  |  |
|  | Error | 489 |  |  | 493 |  |  |  |  |  |
| SEE | Species | 72 | 2.46 | < 0.0001 |  |  |  |  |  |  |
|  | Census method | 1 | 18.81 | < 0.0001 |  |  |  |  |  |  |
|  | Sampling effort | 1 | 1.88 | 0.17 |  |  |  |  |  |  |
|  | Density-dependence | 1 | 1.09 | 0.30 |  |  |  |  |  |  |
|  | Number of years | 1 | 11.06 | 0.00095 |  |  |  |  |  |  |
|  | Habitat fragmentation | 1 | 0.20 | 0.65 |  |  |  |  |  |  |
|  | Latitude | 1 | 0.68 | 0.41 |  |  |  |  |  |  |
|  | Marginality | 1 | 14.57 | 0.00015 |  |  |  |  |  |  |
|  | Error | 489 |  |  |  |  |  |  |  |  |

Species (random factor), census method (fixed factor), sampling effort, density-dependence, number of years surveyed, habitat fragmentation, population latitude and marginality were included in the models as independent variables. In all four cases the full model was highly significant (*F* ≥ 2.71, df = 75,493 or df = 79,489, *r*^2^ ≥ 0.298, *P* < 0.0001).
