# Supplementary material for: Temporal Variation in Population Size of European Bird Species: Effects of Latitude and Marginality of Distribution
Source: PLoS One. 2013 Oct 17;8(10):e77654. doi: 10.1371/journal.pone.0077654 (PMC3798344; doi:10.1371/journal.pone.0077654)

**Appendix S2.** An illustrative example of calculation of population parameters for a common bird: the great tit

In order to make calculation of population parameters easier to understand, we will briefly explain these calculations for a common bird, the great tit (*Parus major*). Great tits breed in all 12 countries included in the present study, and the number of years for which population indices were available ranged from seven (Poland, 2000-2006) to 34 (Sweden, 1975-2008). Population indices for Polish great tits, for example, were 1.00 (2000), 1.01 (2001), 0.82 (2002), 0.93 (2003), 0.95 (2004), 0.95 (2005), and 0.92 (2006). CV of these seven values is 6.87 and CV corrected for sample size is 7.12 (see transformation in section Confounding factors). As another example, population indices for Austrian great tits were 1.00 (1998), 1.02 (1999), 1.02 (2000), 0.99 (2001), 0.99 (2002), 1.03 (2003), 1.15 (2004), 1.04 (2005), and 1.04 (2006). CV of these nine values is 4.78 and CV corrected for sample size is 4.91. Calculations of CV of population indices (corrected for sample size) were done in a similar way for the other ten countries, giving values from 4.91 (Austria) to 18.26 (Finland) (Fig. S1(A)).

Regression of population indices on years allowed us to calculate both the slope and the SEE for great tits in each country. In the case of Poland, the regression equation was y = 18.0362 - 0.0085 x (*r*^2^ = 0.082, SEE = 0.068, *F*_1,5_ = 0.44, *P* = 0.53). In the case of Austria, the regression equation was y = -16.5382 + 0.0088 x (*r*^2^ = 0.239, SEE = 0.046, *F*_1,7_ = 2.20, *P* = 0.18). For great tits in the 12 studied countries, SEE ranged from 0.046 (Austria) to 0.137 (Sweden), and slopes ranged from -0.0088 (Denmark) to 0.0273 (Hungary) (Figs. S1(B) and S1(C)).

Regarding the estimation of density-dependent effects on among-year variability in population size, we divided the population indices for each population in two halves, one with the largest population indices and the other with the smallest population indices. In the case of Poland, the largest indices were 1.01, 1.00, 0.95 and 0.95, and the smallest indices were 0.95, 0.93, 0.92 and 0.82. There were an odd number of study years, and the mid-point population index (0.95) was included in both halves. In the case of Austria, the largest indices were 1.15, 1.04, 1.04, 1.03 and 1.02, and the smallest indices were 1.02, 1.02, 1.00, 0.99 and 0.99. Here there were also an odd number of study years, and the mid-point population index (1.02) was included in both halves. For Poland, CV_high_ was 3.34 and CV_low_ was 6.60 (3.55 and 7.01 respectively when corrected for sample size). For Austria, CV_high_ was 5.07 and CV_low_ was 1.60 (5.32 and 1.68 respectively when corrected for sample size). Following the equation log_10_CV_high_ - log_10_CV_low_, density-dependence was -0.30 for the Polish population and 0.50 for the Austrian population. The procedure was identical for the other ten countries.

To explain the calculation of marginality, we will choose the northernmost (Finland; latitude index = 64.95º) and southernmost (Spain; latitude index = 39.90º) countries in our study. The northernmost and southernmost limits of the breeding distribution of great tits are 70.67º and -9.00º (9.00º South), respectively, with the mid-point between these extremes being 39.84º. The smallest distance between the latitude index of the country and the distribution range limits of the species was 5.72º for Finland and 30.77º for Spain (parameter “L”). The distance between the latitude index of the country and the mid-point between the northernmost and southernmost limits of the breeding distribution was 25.11º for Finland and 0.06º for Spain (parameter “C”). Following the equation log_10_(C+1) - log_10_(L+1), the marginality of the Finnish population was 0.59 and the marginality of the Spanish population was -1.48. Transformation of these values (see transformation in section Population Latitude and Marginality) to ensure that marginality estimates ranged from 0 (central population) to 1 (marginal population) provided the values 0.65 and 0.00 for Finland and Spain, respectively.

**Figure S1. Population parameters for the great tit (*Parus major*) in 12 European countries.** (a) Coefficient of variation (CV) of population size estimates (population indices) corrected for sample size (see text); (b) slope and (c) standard error of the estimate (SEE) after regressing population indices on year.


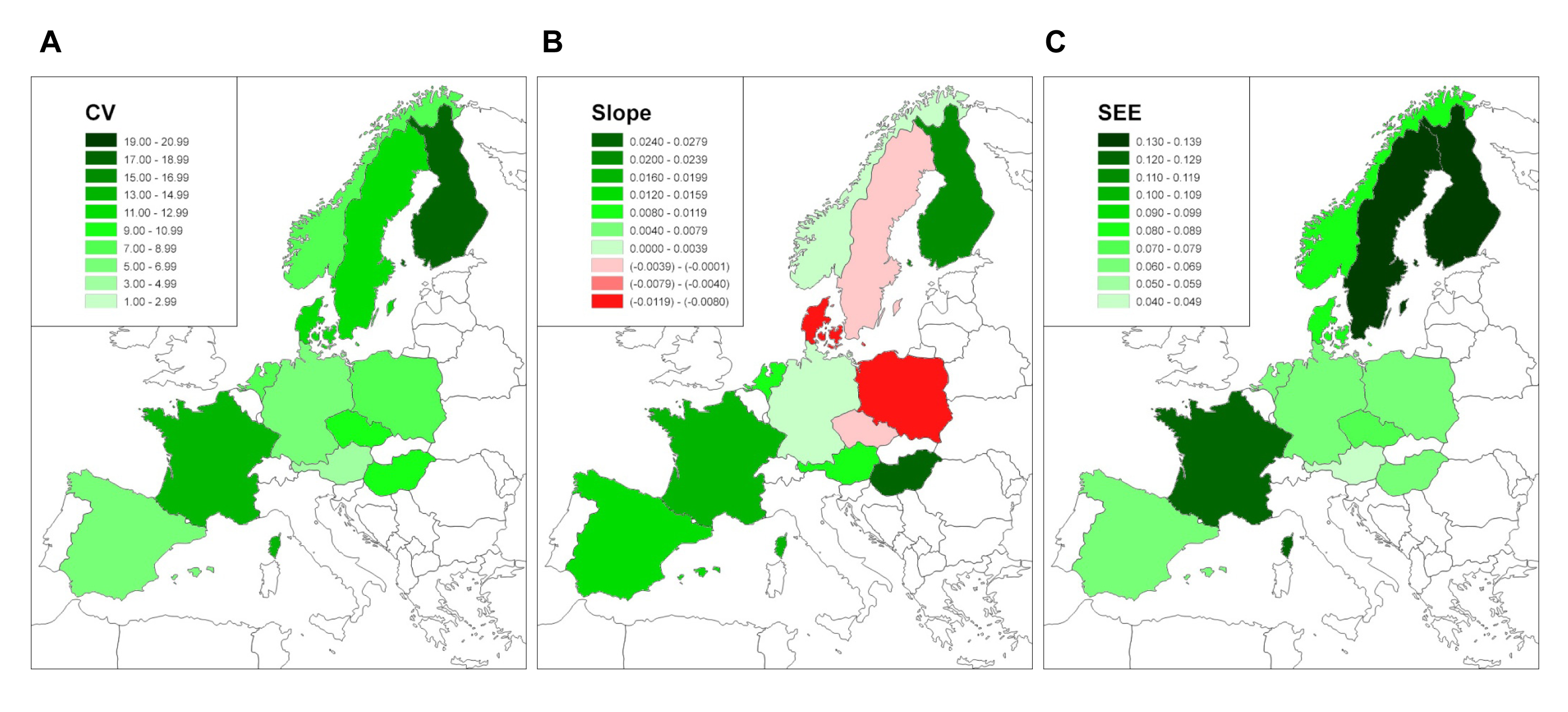

Supplement: Appendix S2 — An illustrative example of calculation of population parameters for a common bird: the great tit. (DOC) [file pone.0077654.s002.doc]
